# Body mass is associated with hibernation length, body temperature, and heart rate in free-ranging brown bears

**DOI:** 10.1186/s12983-023-00501-3

**Published:** 2023-08-17

**Authors:** Alina L. Evans, Boris Fuchs, Navinder J. Singh, Alexandra Thiel, Sylvain Giroud, Stephane Blanc, Timothy G. Laske, Ole Frobert, Andrea Friebe, Jon E. Swenson, Jon M. Arnemo

**Affiliations:** 1 Department of Forestry and Wildlife Management, Faculty of Applied Ecology and Agricultural Sciences, Inland Norway University of Applied Sciences, Campus Evenstad, 2418 Elverum, Norway; 2 Department of Wildlife, Fish and Environmental Studies, Faculty of Forest Sciences, Swedish University of Agricultural Sciences, 901 83 Umeå, Sweden; 3 Reseach Institute of Wildlife Ecology, Department of Interdisciplinary Life Sciences, University of Veterinary Medicine, Savoyenstraße 1, 1160 Vienna, Austria; 4 Hubert Curien Multidisciplinary Institute, UMR 7178 CNRS/UDS, 23 Rue Becquerel, 67087 Strasbourg, France; 5 Department of Surgery, University of Minnesota, Minneapolis, MN 55455 USA; 6 Department of Cardiology, Faculty of Health, Örebro University, Örebro, Sweden; 7 Norwegian Institute for Nature Research, 7485 Trondheim, Norway; 8 Faculty of Environmental Sciences and Natural Resource Management, Norwegian University of Life Sciences, Post Box 5003, 1432 Ås, Norway; 9 Energetics Lab, Department of Biology, Northern Michigan University, Marquette, USA

**Keywords:** Brown bears, Hibernation, Heart rate, Metabolic rate, Thermal conductance, Thermoregulation

## Abstract

**Background:**

Despite centuries of research, debate remains on the scaling of metabolic rate to mass especially for intraspecific cases. The high variation of body mass within brown bears presents a unique opportunity to study the intraspecific effects of body mass on physiological variables. The amplitude of metabolic rate reduction in hibernators is dependent on body mass of the species. Small hibernators have high metabolic rates when euthermic but experience a drastic decrease in body temperature during torpor, which is necessary to reach a very low metabolic rate. Conversely, large hibernators, such as the brown bear (*Ursus arctos*), show a moderate decrease in temperature during hibernation, thought to be related to the bear’s large size. We studied body mass, abdominal body temperature, heart rate, and accelerometer-derived activity from 63 free-ranging brown bears (1–15 years old, 15–233 kg). We tested for relationships between body mass and body temperature, heart rate, and hibernation duration.

**Results:**

The smallest individuals maintained lower body temperatures during hibernation, hibernated longer, and ended hibernation later than large bears. Unlike body temperature, winter heart rates were not associated with body mass. In summer, the opposite pattern was found, with smaller individuals having higher body temperature and daytime heart rates. Body mass was associated with body temperature in the winter hypometabolic state, even in a large hibernating mammal. Smaller bears, which are known to have higher thermal conductance, reached lower body temperatures during hibernation. During summer, smaller bears had higher body temperatures and daytime heart rates, a phenomenon not previously documented within a single mammalian species.

**Conclusion:**

We conclude that the smallest bears hibernated more deeply and longer than large bears, likely from a combined effect of basic thermodynamics, the higher need for energy savings, and a lower cost of warming up a smaller body.

**Supplementary Information:**

The online version contains supplementary material available at 10.1186/s12983-023-00501-3.

## Background

The relationship between body mass and body size between species has been described and studied for over a century [1]. The influence of body size on body temperature (T_b_) and metabolic rate is well established across a variety of classes of vertebrates, including birds, amphibians, and reptiles [2]. When plotted against body mass, the metabolic rates across species, especially for mammals and birds, follow a logarithmic curve [3]. Clarke and Rothery 2008 reviewed data from 596 mammalian species and concluded that body mass, body temperature and metabolic rate are in a complex relationship, mediated through ecology [4]. Morrison and Ryser [5] clearly illustrated the effect of body size on T_b_ across a range of mammalian species from 0.001 to 100,000 kg. However, more recent studies illustrate the difficulty of using one model to predict metabolic rate across all mammalian species [6]. Different scaling exponents, ranging from 0.35 to 0.97, have been reported, for example, for the brown rat (*Rattus norvegicus*) [7]. A meta-review by Glazier 2005 concluded that metabolic scaling is complex and results from adaptations to physiochemical and ecological constraints [8]. Interspecific metabolic scaling in particular differs in regard to temperature regulation, body-size range and activity level. Although minimum T_b_ during hibernation increases with increasing body mass [9], no such relationship has been found for mammals during euthermia [10].

In many animal species, metabolic rate is tightly connected to heart rate (HR) and can be calibrated to metabolic rate with specific validation. Without specific validation, HR can only be used as a qualitative proxy for metabolic rate. The correlation between HR and body mass has been studied in several mammalian species with contrasting results. For example, no difference was found in comparing HR across different sizes of dogs [11, 12], although a study on horses and ponies found body weight to be a strong predictor of HR [13] and a recent study on Canadian lynx (*Lynx canadensis*) found higher HR in smaller individuals compared to larger ones [14]. Whereas in humans, age seems to be a strong predictor for intrinsic heart rate, declining with increasing age [15].

In the literature on mammalian hibernation the question of how mammals of different sizes, ranging from bats to bears, can hibernate and how an animal’s body size affects its status as a hibernator, has been a topic of interest for many years [16, 17]. Most studies on small mammals, which have regular arousals during hibernation (accounting for 72% of the total energy used in marmots during winter [18]), have focused on the effects of pre-hibernation body condition and energy (fat) reserves on subsequent winter hibernation. An interspecific comparison found no relationship between body size and length of torpor bouts across 76 species of hibernators [9]. It is also well accepted that body size determines the degree of reduction in metabolic rate between euthermic and torpid periods; with small hibernators having a higher metabolic rate when euthermic and demonstrating a drastic decrease in metabolic rates to reach low T_b_ in torpor [18]. Some studies have concluded that fatter individuals, i.e. with greater energy reserves, have a higher mean minimum body temperature and arouse more often, and for longer periods, than leaner animals during hibernation [19, 20]. This is likely to reduce the associated somatic costs of torpor (such as oxidative stress, reduced immunocompetence and neuronal damage[21]), indicating intraspecific variation of energetics during hibernation. In contrast, other studies [9, 22–25] have not found evidence of this.

In large hibernating species, such as brown bears (*Ursus arctos*), body mass and body condition (an index of body fat storage) are not related[26]. Body mass has instead been found to correlate with age in brown bears [27, 28]. Few hibernators span such a wide range of body mass as the brown bear, with spring body mass in Scandinavian brown bears ranging from 8 to 44 kg at one year of age [29] to 62–241 kg as adults [30]. This is a life-history trait considerably different from small hibernators, such as the Syrian golden hamster (*Mesocricetus auratus*) at 102–149 g [31], or the Alpine woodchuck (*Marmota monax*), which ranges from 2 to 5 kg from juvenile to adult [32]. The brown bear, with a range of body masses (reflecting body sizes) differing more than 30 times, is therefore deserving of special attention, providing the unique opportunity to study intraspecific variation of hibernation status in relation to body size.

The metabolic rate reduction during hibernation in brown bears, is likely similar to the 75% reduction reported in American black bears [33], which is in contrast to a 95% decrease on average in some small (< 5 kg) hibernators [34]. Based on the reviewed literature, the lower magnitude of temperature fluctuations during hibernation in bears, compared to other hibernators, is thought to be related to the bears’ large body size [18, 35, 36]. Body mass affects thermodynamics and one study in American black bears (*Ursus americanus*) found that larger bears had lower thermal conductance. Although smaller black bears had higher total body conductance, their lower critical temperatures during hibernation was not significantly higher than that of larger bears [37]. The hibernation-optimization hypothesis [38] suggests that hibernators that can afford to spend less time in torpor, and more time at euthermic T_b_ through arousals, reduce the negative effects of rewarming from torpor, including oxidative stress, reduced immunocompetence, and neuronal damage [21]. In this regard, even though bears do not show arousals, larger bears with more fat reserves would be expected to exhibit a shorter hibernation period, as has been previously shown for male brown bears [39], which are generally larger than female bears.

Overall, the large amount of data supporting the well-described interspecific effect of body size on metabolic rates and T_b_ in homeothermic endotherms vs heterothermic endotherms, contrast with the few studies available presenting intraspecific data, in particular for species in which body sizes changes dramatically over their lifespan, such as the brown bear. One study on American black bears (n = 12, body mass 35.5–116.5 kg) in captivity found that smaller bears had higher mass specific metabolic rates during hibernation[37]. So far, the question of whether intraspecific differences in body size are associated with different hibernating and energy saving patterns is understudied.

In this study, we investigated this question using a unique dataset of 63 free-ranging brown bears aged 1–15 years old, having a body mass range from 23 to 233 kg. We tested for relationships between body mass, body temperature and HR in winter and summer, in addition to hibernation duration and timing of emergence from hibernation.

## Materials and methods

### Study area

The study from southcentral Sweden (61°N, 15°E) is part of a long-term individual-based research project on brown bears. The study area covers approximately 13,000 km^2^ and is predominantly covered by intensively managed boreal forest. The altitude gradually increases from ≈150 m above sea level in the east to 850 m above sea level in the west, which is also the approximate tree line. Snow cover increases towards the north-west and with altitude and lasts approximately from late-November to April or May, mean daily temperatures are −7 °C in January and 15 °C in June [40]. During the hyperphagic period, brown bears feed on different berry species (*Vaccinium myrtillus, V. vitis-idea, Empetrum hermaphoditum*) [41]. The study area time zone is 1 h ahead of coordinated universal time (UTC) in winter and 2 h ahead in summer.

### Subjects and devices deployed

**Fig. 1:**
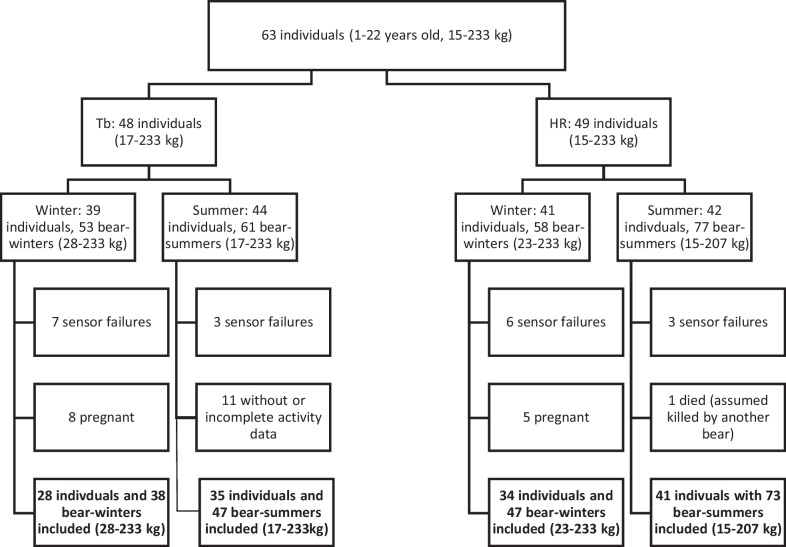
Sample sizes for each analysis. Some brown bears had only body temperature (T_b_) or heart rate (HR) logging, and not both, and some were excluded from HR or T_b_, due to failure of a specific logger or from one season, due to pregnancy, or in one case, killed by another bear. Some bears from summer were not available in winter, because they were killed during the autumn bear hunt

A total of 63 brown bears (1–22 years old, 15–233 kg) were captured by darting from a helicopter, as previously described [42]. A schematic illustration of the data set is presented in Fig. 1 and a detailed overview over all individuals included in the study can be found in Additional file 1: Table S1. Captures were performed from the end of February to early July during 2010–2016. The bears were fitted with global positioning system (GPS) collars (Vectronics Aerospace GmbH, Berlin, Germany), very high frequency (VHF) abdominal implants (Telonics Inc., Mesa, AZ, USA), intraperitoneal temperature loggers (DST Centi, Star Oddi, Gardaber, Iceland), and subcutaneous HR loggers (Reveal XT, Medtronic Inc., Mounds View, Minnesota, USA). At capture, all bears were weighed with a digital spring scale and age was based on known birth year or determined from tooth sections [43]. Bears were captured and weighed in winter (late February to early March), spring (late April to May), or summer (June to early July), depending on the capture period. Bears were weighed every time they were captured and all data for HR and Tb correspond to the same year as body mass measurements. All bears, that were captured in winter were recaptured in summer, bears captured in April/May were typically only captured once that season. We associated winter data with either the body mass at winter capture or from the spring immediately after hibernation, depending on when the bear was first captured. For summer data we used the body mass measured either at summer capture or the preceding spring, depending on when the bear was last captured.

The Reveal XT preprogramed settings provided mean HR from UTC 08:00 am to UTC 08:00 pm (heart rate during daytime, HR_D_) and UTC 00:00 am to UTC 04:00 am (heart rate during nighttime, HR_N_). The heart rate means for each time period were calculated by the device from continuous ECG. These “generation 2” devices are previously described in detail [44]. T_b_ was recorded by the loggers at intervals ranging from 1 to 15 min for 1–3 years. We adjusted the T_b_ measurements to thesame time intervals as the preprogrammed HR measurements to allow for comparison between T_b_ and HR by averaging the T_b_ for night hours (T_bN_) and day hours (T_bD_). This resulted in a time series with two values per day for each physiological variable for each bear. A subset of this data, from 14 bears collected 2010–2012, was included in a study on the drivers of hibernation in brown bears [45].

We divided data on T_b_ and HR into a winter dataset ranging from 1 December to 23 February and a summer dataset from 31 May to 20 August. We chose these dates because, by December, the bear’s T_b_ had reached hibernation levels [45] and in the last week of February, several of the bears were disturbed by capture activities [46, 47]. The summer dataset started on 31 May, because bears have left the den site by that time and are active [45]. We ended the data selection on 20 August, because the legal bear hunting starts on 21 August each year in Sweden, and periods of higher human presence have been reported to affect bear physiology [48] and behavior [49]. Some bears were excluded due to sensor failures or if they were shot in autumn during the bear hunt. We also did not include data from female bears in winter if they were pregnant the same winter because brown bears have elevated T_b_ values during gestation [50]. For clarification and transparency reasons we included a figure to illustrate the process and sample size at initial data collection to final sample size (Fig. 1).

### Accounting for activity during summer

For the active season we chose to filter the T_b_ data to select for inactive periods. This allowed us to compare T_b_ in different sized bears during inactivity, however, we could not compare the results directly to the difference in HR as the aggregation to night and day is fixed by the device. To control for locomotor activity, we merged the raw T_b_ dataset with the accelerometer-based activity data from the GPS collars. The dual-axis motion sensor measures acceleration across two axes with a rate of 6–8 Hz, averages values over 5-min intervals, and generates a numeric value ranging from 0 to 255. We used a previously described threshold value for Scandinavian brown bears to determine whether a bear had been active or inactive during a given 5-min interval (inactivity was when the sum of x and y values were < 50) [51]. We interpolated T_b_ data for each 5-min interval to match the activity data using the base function “approx” in R with the default settings. To exclude active periods from the analysis, we calculated the mean T_b_ for hours with < 2 “active” 5-min intervals, i.e., the bear was active a total of 0–5 min during that hour. For T_b_, we had now accounted for different activity patterns and we no longer analysed day and night separately. Of the 58 complete bear-summers with T_b_ data, we had activity data for 53 (from 37 individual bears). In addition, six bear-summers had to be removed, because of incomplete activity data during the summer, resulting in 47 bear-summers from 35 individual bears. We did not analyse the small-scale activity patterns present during hibernation [52].

### Quantification and statistical analysis

#### Model selection: T_b_ and HR

We fitted generalized additive mixed models (GAMMs) using the function “bam” and the R-packages mgcv [53] and itsadug [54]. We built different models for the following response variables: During winter: T_bD_ and HR_D_ (during daytime); during summer: T_b_ during inactivity, HR_D_ and HR_N_ (daytime and nighttime are defined in methods section “b”). We expected individual, as well as year-to-year, variation and used Aikake information criterion (AIC) to compare the following random structures to account for this variation: (1) random intercept for bear ID, (2) random intercept for the combination of bear and year (further referred to as YID), (3) random intercept for year, (4) random intercept for bear ID nested in year, (5) random intercept and slope for bear ID and (6) random intercept and slope for YID (Additional file 1: Tables S3–S7). We justify the combination of bear and year (YID) by the fast year-to-year growing potential in this population (i.e., even if it is the same bear ID, we consider it statistically to be a different bear). AIC was also used to do model selection on the fixed effects of the models, to compare a (1) null model with a model containing (2) a smoother for day of the year (time) and (3) a tensor product smoother for time, multiplied by the spring body mass (continuous variable), creating an interaction-like term of the two (Additional file 1: Tables S3–S7). This final model was inspected for residual autocorrelation and consequently an autoregressive model (AR1) was added. The autoregression parameter (ρ) was based on the autocorrelation factor of the standardized residuals at lag 1 and then adjusted, based on maximum likelihood values for the same model structure, by varying ρ values (± 0.3). For model interpretation, we predicted T_b_ and HR values for the day in winter for bears with body mass 40 kg and 120 kg. During summer, we predicted HR for day and night and T_b_ during inactivity, also for bears with body mass 40 kg and 120 kg.

**Fig. 2:**
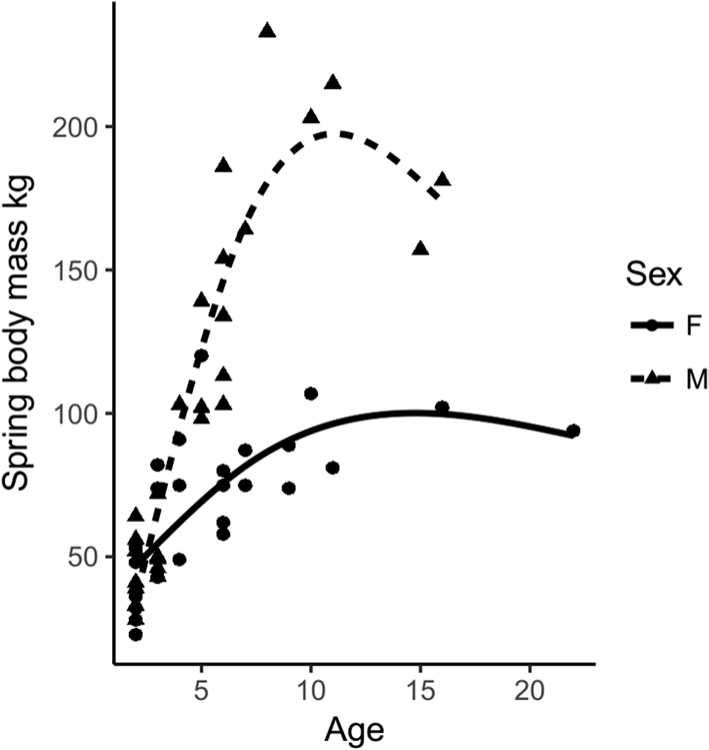
Spring body mass and age for female (dots) and male (triangles) bears from the heart rate dataset. Generalized additive model of the form spring body mass ~ s(age) with a cubic regression spline and basis dimension 3 predicting age based on body mass for females (solid line) and males (dashed line)

Sex, age, and body mass are correlated in this population [27, 28]. Female bears reach asymptotic body mass at 6 years and males reach a larger mass at 12 years [27]. In our dataset, only 13 bears were older than 10 years (Fig. 2) and only six females were heavier than 100 kg. This is due to a high proportion of adult females that either gave birth or den together with offspring and thus were excluded from the data set. To avoid collinearity among the fixed terms, we did not consider age or sex. We excluded age over body mass, because we were more interested in body mass and its effects on T_b_ and HR. As female bears have a lower asymptotic body mass, we first included sex and body mass in an interaction-like term, but biased sample size towards younger and lighter female bears led to overfitting of the models.

**Fig. 3:**
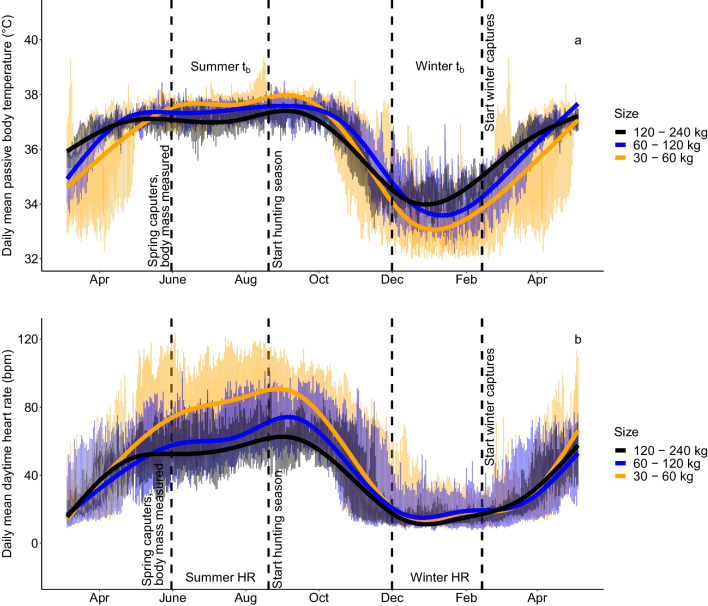
Annual variation in body temperature and heart rate (HR) across size groups; **A** The analysed daily mean body temperature data of brown bears in Sweden measured between 2010 and 2016 pooled in to three body mass groups: Small” (orange) was defined as < 60 kg (N = 25), “medium” (blue) as 60–120 kg (N = 11), and “large” (black) as 120–240 kg (N = 10). **B** Mean daytime HR of brown bears in Sweden measured between 2011 and 2016, pooled in to three body mass groups: “Small” (orange) < 60 kg (N = 32), “medium” (blue) 60–120 kg (N = 31), and “large” (black) 120–240 kg (N = 11). To illustrate the full annual cycle, the same data is partly shown twice (February to June). Sample sizes are for an individual in a given year, body mass measured in either February, May or June

For graphical representation of the raw data sets for T_b_ and HR (Fig. 3), we grouped the bears by size, based on their body mass in the winter/spring, in contrast to the models, where actual body mass was used. “Small” was defined as < 60 kg, “medium” as 60–120 kg, and “large” as 120–240.

#### Model selection: hibernation phenology

We determined the length of the hibernation period from the winter T_b_ dataset. For the start and end of hibernation, we used the first day that the daily mean T_b_ decreased below 36.4 °C for at least 7 consecutive days in autumn and the first day above 36.7 °C when remaining at this threshold for at least 7 consecutive days in spring. These temperatures have been documented to be associated with hibernation start (36.47 ± 0.14 °C) and end (36.70 ± 0.15 °C) [45]. T_b_ data during hibernation start and end in the following spring was available during 35 bear winters over 4 years from 25 individual bears, 12 females and 13 males, ranging in body mass from 30 to 233 kg. We also expected individual, as well as year-to-year, variation regarding the bears’ hibernation phenology and tried to fit linear mixed models with bear ID and year as random components. Aikake information criterion (AICc) differences between model fitted with and without year or bear ID was exactly 2, suggesting no change in deviance, i.e. no information gain by using a random component. Both year and bear ID showed very little variation across all years and we decided not to fit any random component. We proceeded using generalized linear models with a Poisson error distribution and a logarithmic link function for three different response variables: hibernation length in days, the start of hibernation as day of the year, and the end of the hibernation as day of the year. For each response, we selected a set of a priori formulated candidate models. Some bears were recaptured during hibernation and, as this might have led to a delayed end of hibernation [37], we included winter capture as a categorical factor to the model selection for hibernation length and hibernation end; but not for start of hibernation. Further we included a factor for the year and spring body mass. The candidate model set contained a null model and all possible variable combinations. All models were compared to each other and to a null model using AICc (Additional file 1: Table S10). We averaged all models within Δ AICc 2 using model averaging from the AICcmodavg package [55]. We performed all analyses using statistical extensions available in R 3.4.2 [56].

## Results

### Model performance

**Fig. 4:**
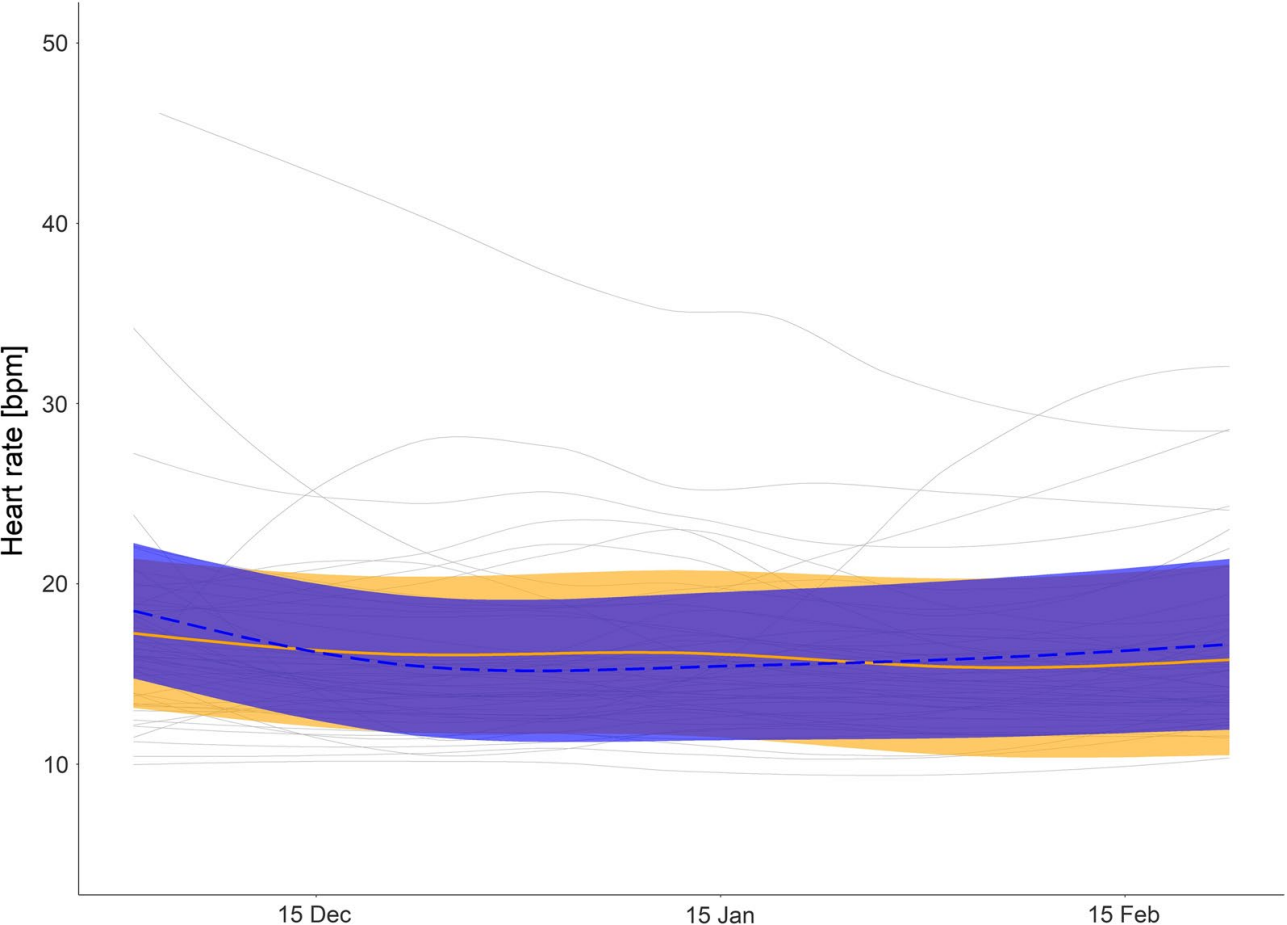
Heart rate (HR) in winter: Predicted mean daytime HR for hibernating brown bears with body mass of 40 kg (orange solid line), bears with body mass of 120 kg (blue dashed line), and mean smoothed values for each bear winter (grey solid line). Shaded areas display 95% confidence intervals. HR data from 1 December to 23 February

**Fig. 5:**
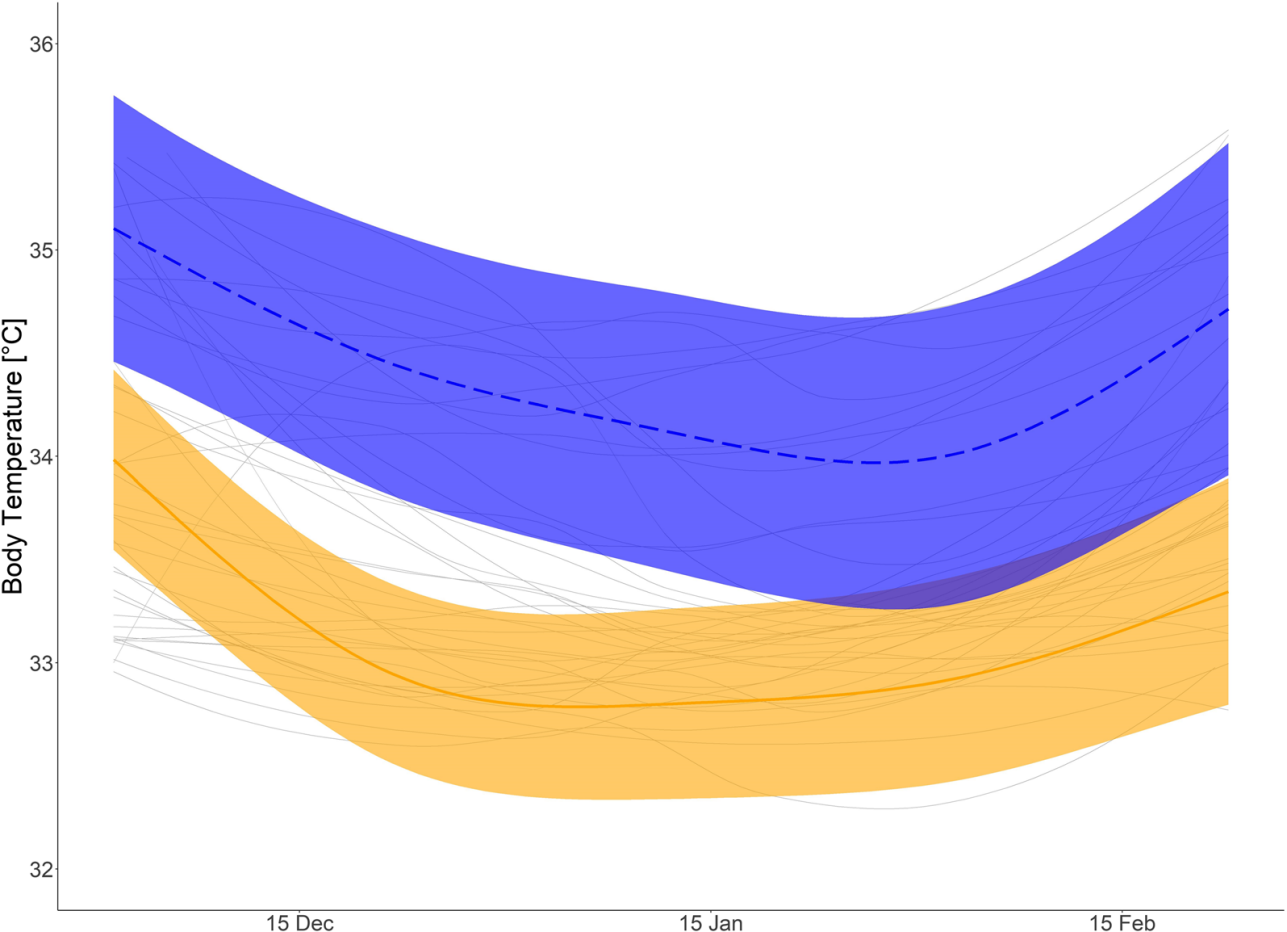
Body temperature in winter: Predicted mean daytime body temperature (T_b_) for hibernating brown bears with body mass of 40 kg (orange solid line), and bears with body mass of 120 kg (blue dashed line) and mean smoothed values for each bear winter (grey solid line). Shaded areas display 95% confidence intervals. T_b_ from 1 December to 23 February

**Fig. 6:**
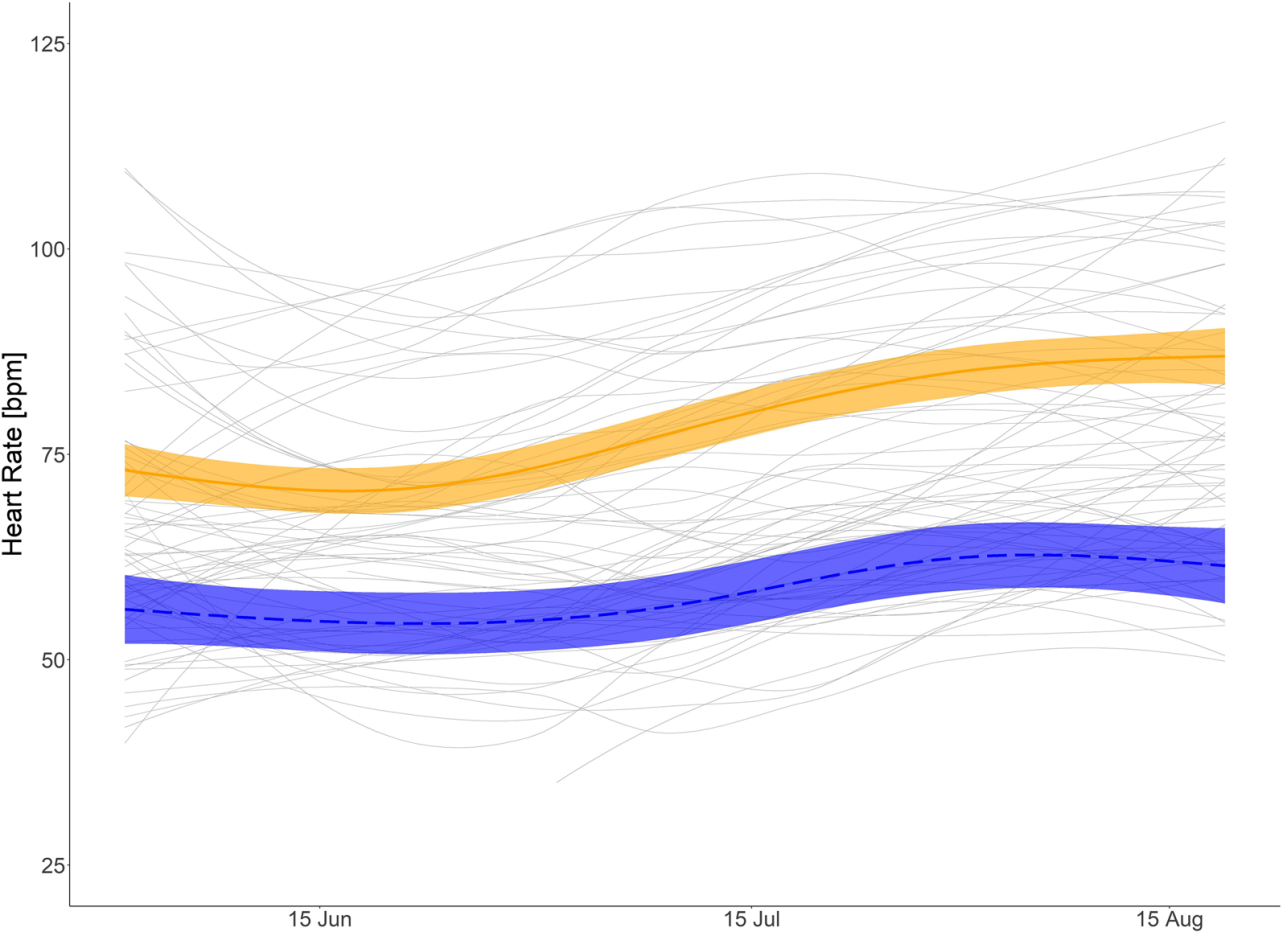
Daytime summer heart rate (HR): Predicted mean HR (HR) for brown bears during summer and during day time with body mass of 40 kg (orange solid line), and bears with body mass of 120 kg (blue dashed line) and mean smoothed values for each bear summer (gray solid lines). Shaded areas display 95% confidence intervals. HR data from 1 June to 21 August measured between 10:00 and 22:00

**Fig. 7:**
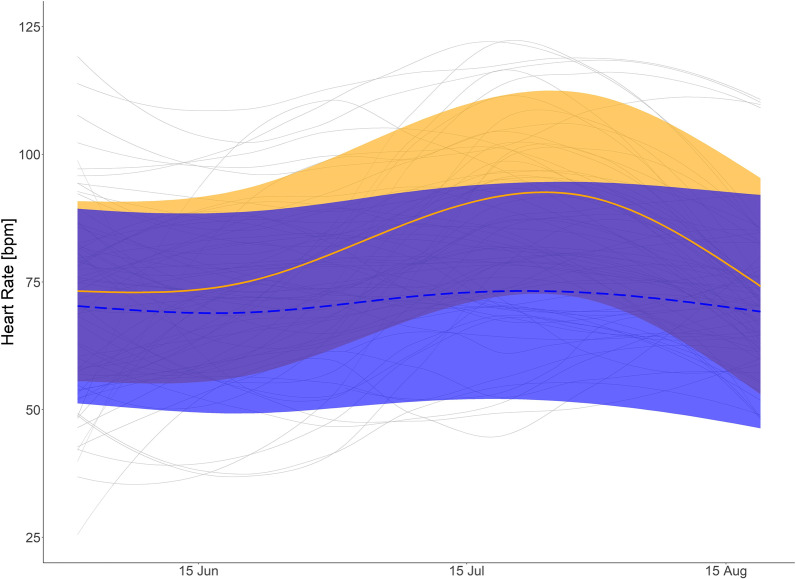
Nighttime summer heart rate (HR): Predicted mean HR (HR) for brown bears during summer and at night with body mass of 40 kg (orange solid line), and bears with body mass of 120 kg (blue dashed line) and mean smoothed values for each bear summer (grey solid line). Shaded areas display 95% confidence intervals. HR data from 1 June to 20 August measured between 02:00 and 06:00

**Fig. 8:**
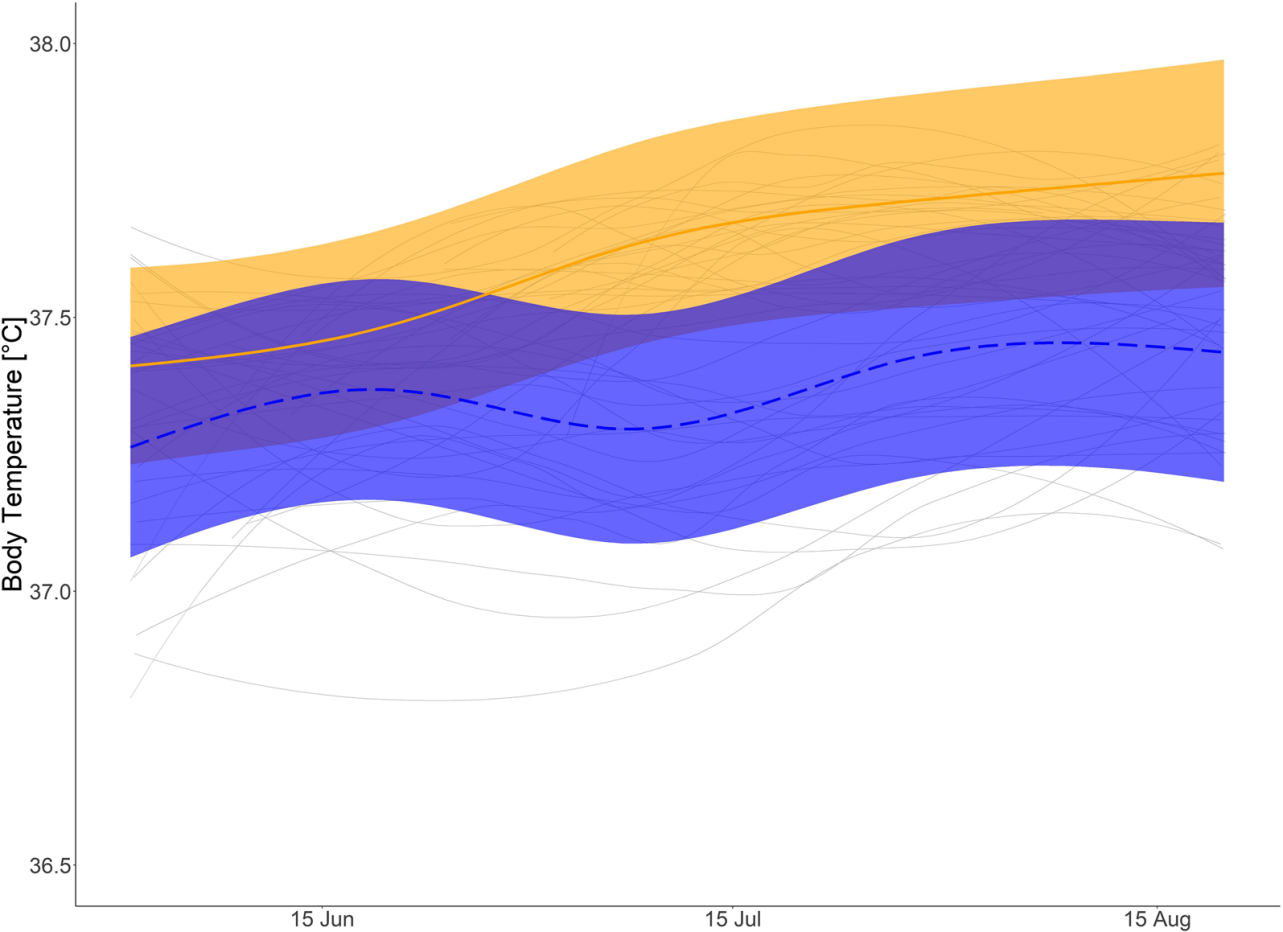
Passive summer body temperature: Predicted daily mean body temperature (T_b_) for brown bears during inactivity in summer with body mass of 40 kg (orange solid line), and bears with body mass of 120 kg (blue dashed line) and mean smoothed values for each bear summer (grey solid line). Shaded areas display 95% confidence intervals. T_b_ data from 1 June to 21 August

For all GAMM models, the highest ranked models included a random intercept and slope for YID (Additional file 1: Table S3–S7, model outputs provided in Additional file 1: Table S8). This led to varying modelled values over time, which had to be interpreted graphically. To better illustrate the predicted differences between small and large bears, only model predictions of bears of 40 and 120 kg are presented in Figs. 4, 5, 6, 7 and 8. Residual autocorrelation was high and AR 1 structures were included in all models, which did not eliminate, but considerably lowered, the autocorrelation to acceptable levels (Figure S1a–e).

### Winter

#### Hibernation physiology

Body temperate and HR varied on a seasonal scale, dependent on body mass (Fig. 3, Additional file 1: Table S2, Figure S2). However, the model did not predict an effect of body mass on average daytime HR during hibernation (Fig. 4), but it did predict that larger bears had a higher overall daytime T_b_ with increasing differences in the start and again towards the end of the hibernation period (Fig. 5). For both, HR and Tb during hibernation, the models including the interaction of day of the year and body mass were ranked highest in the model selection process (Additional file 1: Table S3 and S4), which suggests that the inclusion of body mass improved model fit.

Of the 4,165 daytime HR values, a total of 38 values (0.9%) were above 37 bpm (mean HR at den entry [45]) and 32 of these (0.8%) were from one individual. The mean daytime HR was 16 bpm (SD 5.6 bpm), ranging from 7 to 70 bpm. The mean daytime T_b_ during winter was 33.6 °C, ranging from 30.2 to 37.3 °C (SD 1.0 °C).

#### Start of hibernation

Bears entered the den on average on the 30th of October (range: 10th October–16th November). Spring body mass did not explain the variation in the initiation of hibernation. The null model was the top model with den entry as response variable, indicating that there was no difference in the timing of den entry between different body sizes (Additional file 1: Table S9).

#### Hibernation duration

Larger bears displayed shorter hibernation periods (indicated by an earlier den exit). For example, for bears with a spring body mass of 120 kg, the model predicted 155 days in hibernation (95% ci 133–177 days), but for bears of 40 kg the model predicted 166 days (95% ci 152–186 days). The effect of spring body mass was negative (95% ci not overlapping with 0), indicating a decrease in hibernation period with increasing body mass. Winter capture and sex had no clear effect. Models including spring body mass alone or with winter capture in addition, were all within Δ AICc 2 (Additional file 1: Table S10), did not perform substantially differently and were therefore averaged for prediction. For predictions and interpretation, we used females that had not been captured in winter.

#### End of hibernation

Bears exited the den on average on the 10th of April (range: 15th March–3rd May). For the timing of the end of hibernation, the models with both body mass and winter capture as fixed factors were within AICc delta 2 i.e. no clear difference in model performance (Additional file 1: Table S11). There was a strong effect of spring body mass, but not of winter capture (see ci of average model, Table 1). Larger bears (not captured) raised their T_b_ earlier over the T_b_ level defined as hibernation (Table 1). For example, the model predicted that bears of 120 kg end hibernation around April 1 (day of the year 93; 95% ci 77–111), whereas bears of 40 kg end hibernation around April 14 (day of the year 104; 95% ci 92–115). The same model predicted a 7-day (95% ci 1–17 days) delay in the end of hibernation if a bear of 40 kg was captured in the den.

**Table 1:** Hibernation duration and hibernation end

	Estimate	SE	Lower 95% ci	Upper 95% ci
Hibernation duration				
Intercept	5.16	0.03	5.09	5.22
Spring body mass kg	−1.02(10^–3^)	0.31(10^–3^)	−1.61(10^–3^)	−0.41(10^–3^)
Winter captured	0.05	0.03	−0.02	0.11
*Degrees of freedom: 32*				

Hibernation end				
Intercept	4.70	0.04	4.61	4.78
Spring body mass kg	−1.37(10^–3^)	0.41(10^–3^)	−2.18(10^–3^)	−0.57(10^–3^)
Winter captured	0.06	0.04	−0.01	0.14
*Degrees of freedom: 32*				

Averaged generalized linear model output of the models predicting the duration of hibernation or end of hibernation of brown bears including the variables spring body mass and whether bears were captured in winter. Models are fitted with a logarithmic link function and estimates need to be exponentiated to get days in hibernation (for hibernation duration) or the day of the year (for hibernation end)

### Summer

During the day in summer, HR was lower in larger bears. During night, however, HR was similar among bears. The highest ranked models on the fixed effects structures for day and nighttime HR as well as passive T_b_ during summer included the interaction like term for day of the year and body mass (Additional file 1: Table S5–S7) indicating that there those parameters differ between body masses (see also Additional file 1: Table S2). Visual interpretation of the prediction graph of the HR model for a 120 kg and 40 kg bear indicate decreased HR during the day for larger bears, whereas a 40 kg bears would remain at a similar HR during day and night (Figs. 6 and 7). Predicted values for daytime HR had smaller confidence intervals than nighttime values (Figs. 6 and 7) Inactive T_b_ in summer was lower in larger than smaller bears (Fig. 8). In the beginning of summer, Tb of small and large bears were comparable but followed different trends throughout the course of summer with smaller bears having higher T_b_ than larger bears.

## Discussion

We evaluated the effect of body mass on T_b_ and HR of brown bears in Scandinavia throughout their annual cycle. The smallest individuals reached lower T_b_ during hibernation, hibernated longer, and ended hibernation later than large bears. In contrast to T_b_, HR in winter was not associated with body mass. These relationships were consistent across a range of body masses (15–233 kg). In summer, we observed the opposite pattern, with smaller bears exhibiting higher daytime HR and trending towards higher T_b_. As the smallest bears (cubs still with their mothers) move the least [57], this cannot be explained by activity. We also found that body mass played an important role in some of the phenological aspects of hibernation. Although body mass did not influence date of den entrance, the smallest bears ended hibernation latest and therefore hibernated longer in total. The finding that body mass did not have an effect on den entrance date, is consistent with our previous finding that den entrance timing is primarily dependent on environmental cues [45].

### Winter

The smaller bears had higher T_b_ in summer, but as they entered hibernation, the relationship between T_b_ and body size was reversed. Interestingly, this pattern was not seen for HR, which was similar across sizes (Fig. 3, Additional file 1: Figure S2, Additional file 1: Table S2). Similar HRs in winter could indicate similar hibernating metabolic rates across sizes. These results demonstrate that the difference in T_b_ is not dependent on heart rate and more likely attributed to the fact that smaller animals have a higher surface-to-volume ratio, resulting in higher thermal conductance [37] and poorer heat conservation [34]. This higher thermal conductance in the smaller bears results in their ability to reduce Tb to a greater extent, but HR remains similar because the range in Tb may not be large enough to cause in a difference in HR between groups. This is consistent with interspecies comparisons, where larger hibernators have slower cooling rates [34], confirming that differences can be attributed to body size, even intra-specifically. In contrast to a previous study [37], which found that captive American black bears had a higher mass-specific minimum metabolic rate, smaller brown bears did not have higher HR, but rather similar HR to larger bears, indicating a similar metabolic rate (unfortunately that study did not look at HR, and we have not measured metabolic rate). Interestingly, the smaller bears in our study did not compensate for the increased heat loss by increasing HR and thus metabolic rate. Alternatively, there could be higher MR costs for small bears due to their high thermal conductance, raising their HR to the level of the larger bears. An interesting further question would be if the extreme sinus arrythmias are more common in smaller bears.

We noted that the smallest bears exit their dens last, with several possible explanations; 1) they have smaller fat reserves [60] and must hibernate longer to conserve energy until food availability increases. In contrast to larger bears which have larger fat reserves [60], allowing them to better withstand harsh weather and search for rare, protein-rich food, such as ungulate carrion or weakened moose (*Alces alces*) [39, 41, 61]., or 2) their higher critical temperature (T_A_) is higher than that of the larger bears, so they need a higher ambient temperature before being triggered to emerge in spring (ambient temperature is a driver of den emergence [62]).. However, a study examining the lower critical temperature across black bear body masses of 40–120 kg did not find a significant effect of body mass on lower critical temperature, possibly because of the smaller number of bears or alternatively the higher minimum metabolic rate in smaller hibernating bears might compensate for the increased conductance [37]. This contrasts to our finding that heart rate was not determined by body size during hibernation.

The difference in T_b_ between the larger and smaller bears also did not increase during the duration of the winter, which would be expected if the differences were purely due to increased thermal conductance and heat loss in smaller bears. HR did not differ and was stable between the two groups throughout the winter, even as T_b_ was dropping. This indicates that no active compensation was occurring. Most likely, the smaller bear’s later emergence is due to a combination and interaction between the factors discussed here.

### Summer

In summer, body mass also influenced the T_b_ of inactive bears, and the effect seemed to increase towards the end of summer with smaller bears having higher T_b_ than larger bears. Our models predicted smaller bears to have higher HR than larger bears during the day. Smaller bears had similar predicted HRs between night and day. The larger bears increased their HR in the night, supporting similar HRs at night among bear sizes. Smaller bears have previously been shown to be more nocturnal than larger individuals [63], perhaps their persistent high HR is because of higher activity levels than the larger bears during daytime. An additive factor could be that, although they were foraging more at night, the small bears still needed to remain vigilant at all times, because of the possibility of intraspecific predation [64]. Additionally, day length changes substantially in the study area throughout the year with sunrise as early as 01:30 UTC and the rest of the night is represented by twilight. Brown bears in this study area have been shown to be active during twilight hours in summer [65, 66], which partly corresponds to our night-time HR definition (00:00–04:00 UTC). Consequently, this might have impacted our HR results when comparing day and night HR. The higher T_b_ in smaller bears, despite filtering for inactive hours, indicates a body mass dependent (rather than activity-dependent) difference.

The study had some limitations linked to lack of continuous measurements of body mass throughout the year. We had a rough estimate of body mass when analysing data on a yearly scale. Although sufficient to compare large differences in body mass, analyses on a fine scale of body mass had to be avoided. Thus, weight gain due to the hyperphagia phase in summer could not be accounted for in the analysis. Additionally, the HR analysis was limited by the preprogramed setting on the device, which gave only “day” and “night”, rather than continuous HR data. Settings for 2-min HR are now available [44], but were not available at the time of data collection. We cannot rule out that HR and T_b_ decrease with age and that body mass and age are confounding factors. Distribution of body weights in relation to age is presented in Fig. 7. Also, although these devices have been used with high success in both black bears and brown bears [44], a specific validation of the heart rate calculations would strength both our study and other studies. This is challenging with the current programming of the device to only record episodes of high and low heart rate, however newer models allow for real-time transmission of HR and ECG [44].

Although the literature is rife with interspecific patterns [3, 5], these patterns didn’t necessarily hold true when evaluated within a single species, the Scandinavian Brown bear. Smaller bears had higher T_b_ in summer and lower T_b_ in winter, in line with the principles of thermodynamics and surface-to-volume ratios. The large dataset available here allowed us to conduct comparisons across a range of sizes of free-ranging brown bears, where the relationship between environment and physiology were not altered, as is often the case for captive bears [67]. We conclude that the smallest bears hibernated more deeply and longer than large bears, likely from a combined effect of basic thermodynamics (high thermal conductance, due to a high surface-to-body-mass ratio), the higher need for energy savings, and a lower cost of warming up a smaller body. During summer, smaller bears had higher T_b_ and daytime HR, which is a finding not previously documented within a single mammalian species.

### Supplementary Information

**Additional file 1**. This supplemental material included the referenced material including autocorrelation plots of the models, an overview plot of body temperature and heart rate in relation to body size, overview table of each individual used in the analysis, overview table of monthly average body temperature and heart rate, AIC model selection tables and summary statistics for the highest ranked models.

## Declarations

### Data statement

Data requests should be addressed to Alina L Evans. HR and T_b_ data from brown bears and other species are stored at the Umeå Center for Wireless Remote Animal Monitoring (http://www.slu.se/wram).
