# Supplementary material for: Body mass is associated with hibernation length, body temperature, and heart rate in free-ranging brown bears
Source: Front Zool. 2023 Aug 17;20:27. doi: 10.1186/s12983-023-00501-3 (PMC10433566; doi:10.1186/s12983-023-00501-3)
Supplement: Supplementary file 1 — Additional file 1. This supplemental material included the referenced material including autocorrelation plots of the models, an overview plot of body temperature and heart rate in relation to body size, overview table of each individual used in the analysis, overview table of monthly average body temperature and heart rate, AIC model selection tables and summary statistics for the highest ranked models. [file 12983_2023_501_MOESM1_ESM.docx]

# Supplemental Figures and Tables

**Body mass is associated with hibernation length, body temperature, and heart rate in free-ranging brown bears**

Alina L. Evans^1*^, Boris Fuchs^1*^, Navinder J. Singh^2^, Alexandra Thiel^1^, Sylvain Giroud^3^, Stephane Blanc^4^, Timothy G. Laske^5^, Ole Frobert^6^, Andrea Friebe^7^, Jon E. Swenson^8^, Jon M. Arnemo^1,2^

^1^ Department of Forestry and Wildlife Management, Faculty of Applied Ecology and Agricultural Sciences, Inland Norway University of Applied Sciences, Campus Evenstad, NO-2418 Elverum, Norway

^2^ Department of Wildlife, Fish and Environmental Studies, Faculty of Forest Sciences, Swedish University of Agricultural Sciences, SE-901 83, Umeå, Sweden

^3^ Reseach Institute of Wildlife Ecology, Department of Interdisciplinary Life Sciences, University of Veterinary Medicine, Savoyenstraße 1, 1160 Vienna, Austria

^4^ Hubert Curien Multidisiplinary Institute, UMR 7178 CNRS/UDS, 23 rue Becquerel, 67087 Strasbourg, France

^5^ Department of Surgery, University of Minnesota, Minneapolis, MN 55455 USA

^6^ Department of Cardiology, Faculty of Health, Örebro University, Örebro, Sweden

^7^ Norwegian Institute for Nature Research, NO-7485, Trondheim, Norway

^8^ Faculty of Environmental Sciences and Natural Resource Management, Norwegian University of Life Sciences, Post Box 5003, NO-1432 Ås, Norway

Author for Correspondence: Alina L. Evans; email: [alina.evans@inn.no](mailto:alina.evans@inn.no)

* These authors contributed equally

**Figure S1 -S2**

**Tables S1 – S11**


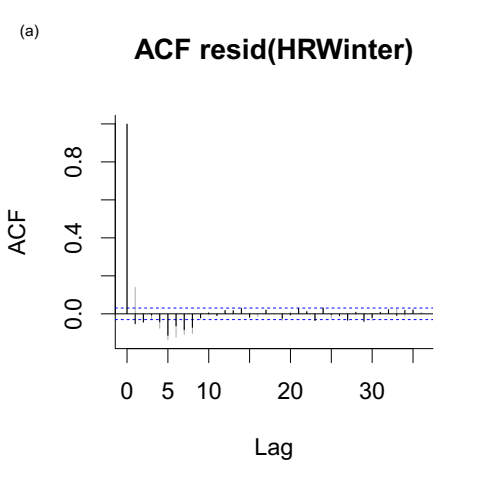

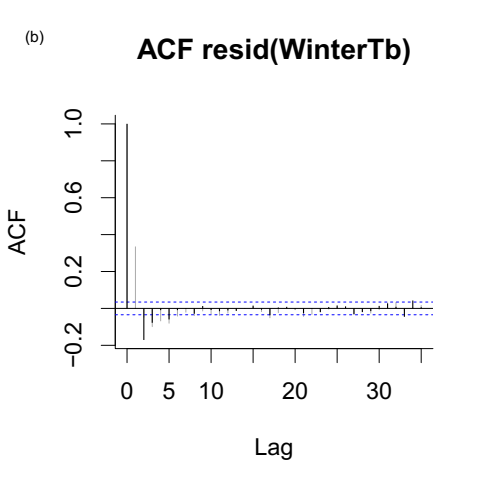


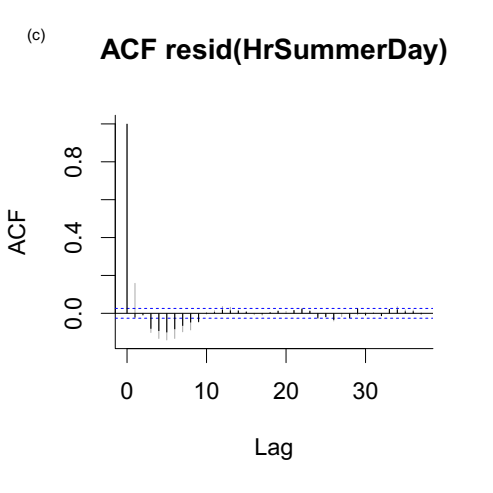

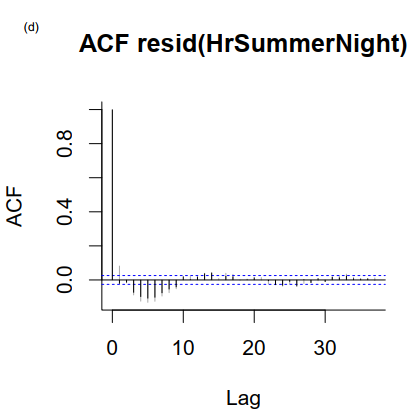


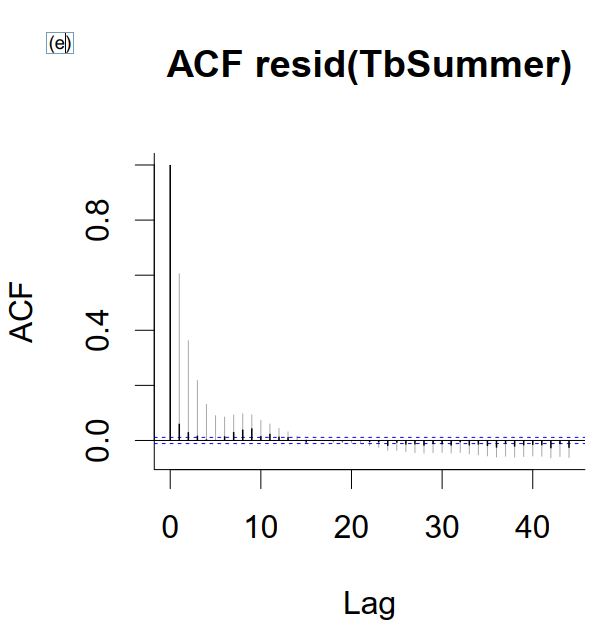


**Figure S1 a-e:** Residual auto correlation depending on lag 2 for the body temperature and the heart rate models. Gray vertical bars indicate residual autocorrelation before implementing the AR 1 structure in to the models and black vertical bar indicate residual autocorrelation with the AR 1 structure.


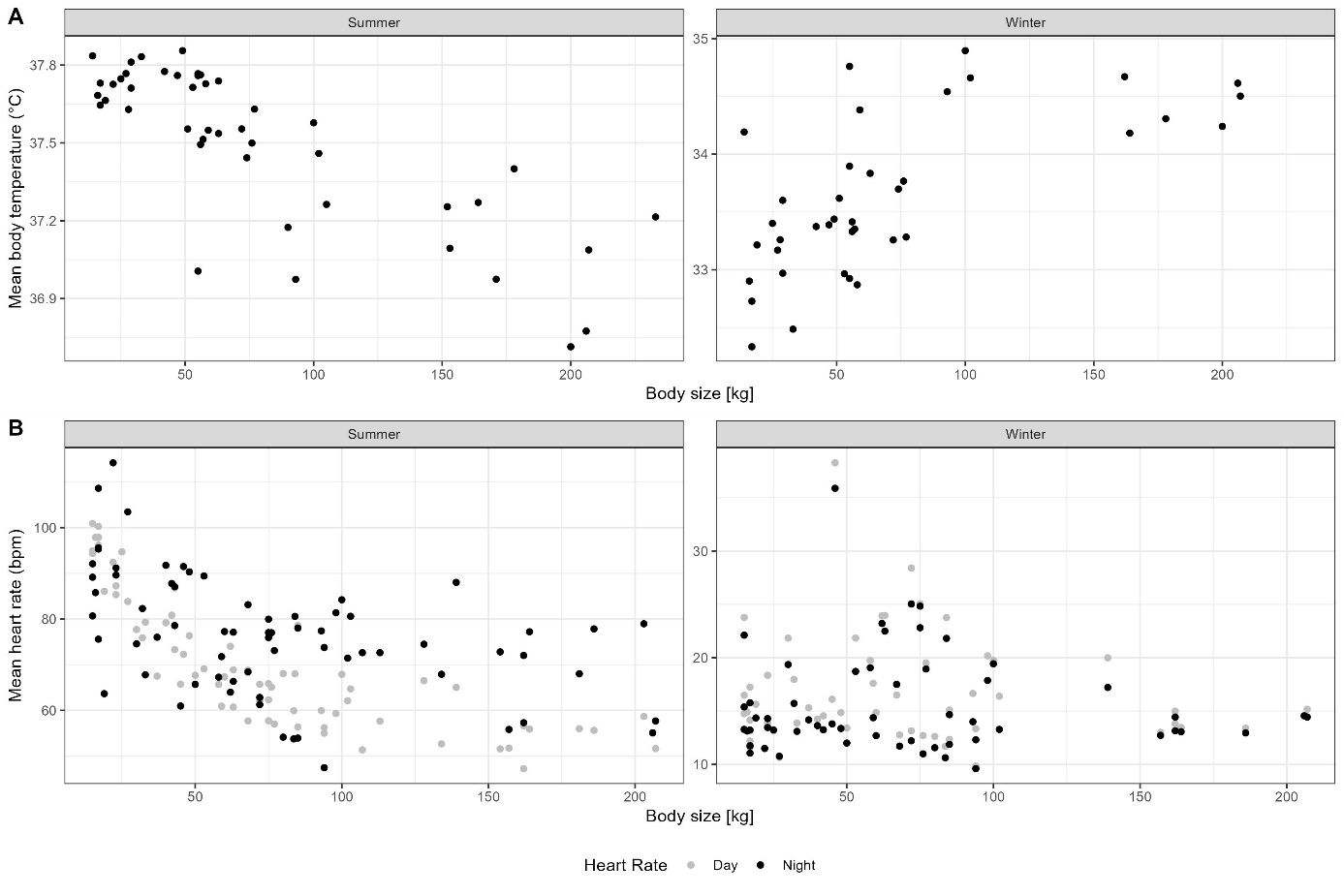


**Figure S2:** A) Mean passive body temperature (°C) during summer and winter for each individual bear in relation to body size (kg). B) Mean daytime (grey) and nighttime (black) heart rate (bpm) during summer and winter for each individual bear in relation to body size (kg).

| **Table S1: Overview over every individual used in the analyses.**  Bear ID = individual bear identification, Year = Year of data collected and used in the analysis for this particular bear, Sex = sex of the bear, Body size = body size in kg for this bear, Age = age of the bear, Date of weight measurement = date when bear was captured and body weight measurement was taken, Period = period which was analysed (summer and/or winter), Data = which type of data was analysed (heart rate and/or body temperarute).  For two individuals we didn’t have the exact capture date but knew it was captured the same year as the data was collected (indicated by only the year in the Date of weight measurement column). | | | | | | | |
| --- | --- | --- | --- | --- | --- | --- | --- |
| **Bear ID** | **Year** | **Sex** | **Body size** | **Age** | **Date of weight measurement** | **Period** | **Data** |
| W0104 | 2015 | F | 83.6 | 15 | 4/24/2015 | Summer | Heart Rate |
| W0605 | 2013 | F | 72 | 8 | 4/22/2013 | Summer | Heart Rate |
| W0605 | 2015 | F | 85 | 10 | 4/24/2015 | Summer | Heart Rate |
| W0610 | 2013 | F | 75 | 8 | 4/19/2013 | Summer | Heart Rate |
| W0611 | 2015 | F | 68 | 10 | 5/16/2015 | Summer | Heart Rate |
| W0620 | 2014 | F | 94 | 9 | 4/27/2014 | Summer | Heart Rate |
| W0620 | 2015 | F | 107 | 10 | 4/18/2015 | Summer | Heart Rate |
| W0625 | 2013 | M | 162 | 10 | 4/18/2013 | Summer | Heart Rate |
| W0625 | 2014 | M | 203 | 11 | 4/23/2014 | Summer | Heart Rate |
| W0805 | 2012 | M | 207 | 10 | 4/19/2012 | Summer | Heart Rate |
| W0806 | 2013 | F | 77 | 6 | 5/22/2013 | Summer | Heart Rate |
| W0806 | 2014 | F | 75 | 7 | 4/23/2014 | Summer | Heart Rate |
| W0811 | 2011 | M | 76 | 4 | 4/15/2011 | Summer | Heart Rate |
| W0811 | 2012 | M | 102 | 5 | 4/19/2012 | Summer | Heart Rate |
| W0811 | 2013 | M | 134 | 6 | 4/17/2013 | Summer | Heart Rate |
| W0812 | 2012 | M | 93 | 5 | 4/19/2012 | Summer | Heart Rate |
| W0812 | 2013 | M | 113 | 6 | 4/16/2013 | Summer | Heart Rate |
| W0820 | 2012 | F | 62 | 5 | 4/27/2012 | Summer | Heart Rate |
| W0825 | 2012 | F | 50 | 5 | 4/24/2012 | Summer | Heart Rate |
| W0825 | 2013 | F | 58 | 6 | 4/19/2013 | Summer | Heart Rate |
| W0904 | 2012 | F | 63 | 4 | 4/26/2012 | Summer | Heart Rate |
| W0908 | 2012 | M | 60 | 4 | 4/20/2012 | Summer | Heart Rate |
| W0908 | 2013 | M | 98 | 5 | 4/16/2013 | Summer | Heart Rate |
| W0908 | 2014 | M | 154 | 6 | 4/16/2014 | Summer | Heart Rate |
| W0910 | 2012 | M | 100 | 4 | 4/20/2012 | Summer | Heart Rate |
| W0910 | 2013 | M | 139 | 5 | 4/17/2013 | Summer | Heart Rate |
| W0910 | 2014 | M | 186 | 6 | 4/16/2014 | Summer | Heart Rate |
| W0910 | 2015 | M | 164 | 7 | 4/18/2015 | Summer | Heart Rate |
| W1011 | 2014 | F | 71 | 5 | 4/24/2014 | Summer | Heart Rate |
| W1017 | 2014 | F | 75 | 5 | 4/25/2014 | Summer | Heart Rate |
| W1017 | 2015 | F | 80 | 6 | 5/13/2015 | Summer | Heart Rate |
| W1019 | 2011 | M | 206 | 7 | 4/15/2011 | Summer | Heart Rate |
| W1103 | 2012 | M | 30 | 2 | 4/25/2012 | Summer | Heart Rate |
| W1205 | 2014 | F | 59 | 3 | 4/15/2014 | Summer | Heart Rate |
| W1206 | 2014 | F | 63 | 3 | 4/15/2014 | Summer | Heart Rate |
| W1206 | 2015 | F | 91 | 4 | 4/16/2015 | Summer | Heart Rate |
| W1206 | 2016 | F | 120 | 5 | 4/30/2016 | Summer | Heart Rate |
| W1210 | 2013 | M | 85 | 5 | 4/17/2013 | Summer | Heart Rate |
| W1210 | 2014 | M | 103 | 6 | 4/16/2014 | Summer | Heart Rate |
| W1211 | 2014 | M | 162 | 14 | 4/17/2014 | Summer | Heart Rate |
| W1211 | 2015 | M | 157 | 15 | 4/17/2015 | Summer | Heart Rate |
| W1211 | 2016 | M | 181 | 16 | 4/27/2016 | Summer | Heart Rate |
| W1301 | 2013 | M | 17 | 1 | 4/22/2013 | Summer | Heart Rate |
| W1302 | 2013 | M | 17 | 1 | 4/22/2013 | Summer | Heart Rate |
| W1302 | 2014 | M | 52 | 2 | 4/24/2014 | Summer | Heart Rate |
| W1303 | 2014 | F | 43 | 2 | 7/2/2014 | Summer | Heart Rate |
| W1304 | 2013 | F | 19 | 1 | 4/23/2013 | Summer | Heart Rate |
| W1304 | 2014 | F | 33 | 2 | 7/2/2014 | Summer | Heart Rate |
| W1304 | 2015 | F | 45 | 3 | 7/7/2015 | Summer | Heart Rate |
| W1306 | 2013 | M | 23 | 1 | 4/29/2013 | Summer | Heart Rate |
| W1307 | 2014 | M | 32 | 2 | 4/23/2014 | Summer | Heart Rate |
| W1308 | 2013 | M | 15 | 1 | 4/29/2013 | Summer | Heart Rate |
| W1308 | 2014 | M | 39 | 2 | 4/23/2014 | Summer | Heart Rate |
| W1314 | 2015 | M | 128 | 6 | 4/16/2015 | Summer | Heart Rate |
| W1315 | 2013 | F | 15 | 1 | 5/22/2013 | Summer | Heart Rate |
| W1316 | 2013 | M | 17 | 1 | 5/22/2013 | Summer | Heart Rate |
| W1316 | 2014 | M | 42 | 2 | 7/1/2014 | Summer | Heart Rate |
| W1316 | 2015 | M | 43 | 3 | 4/22/2015 | Summer | Heart Rate |
| W1317 | 2013 | M | 16 | 1 | 5/22/2013 | Summer | Heart Rate |
| W1317 | 2014 | M | 40 | 2 | 7/1/2014 | Summer | Heart Rate |
| W1317 | 2015 | M | 46 | 3 | 4/22/2015 | Summer | Heart Rate |
| W1401 | 2014 | F | 15 | 1 | 4/26/2014 | Summer | Heart Rate |
| W1401 | 2015 | F | 23 | 2 | 4/22/2015 | Summer | Heart Rate |
| W1403 | 2014 | M | 17 | 1 | 4/26/2014 | Summer | Heart Rate |
| W1404 | 2015 | M | 37 | 2 | 4/23/2015 | Summer | Heart Rate |
| W1404 | 2016 | M | 68 | 3 | 7/6/2016 | Summer | Heart Rate |
| W1407 | 2014 | F | 22 | 1 | 4/27/2014 | Summer | Heart Rate |
| W1407 | 2015 | F | 48 | 2 | 4/18/2015 | Summer | Heart Rate |
| W1408 | 2014 | F | 25 | 1 | 4/27/2014 | Summer | Heart Rate |
| W1408 | 2015 | F | 53 | 2 | 4/18/2015 | Summer | Heart Rate |
| W1409 | 2014 | M | 27 | 1 | 4/27/2014 | Summer | Heart Rate |
| W9403 | 2014 | F | 94 | 21 | 7/6/2014 | Summer | Heart Rate |
| W1301 | 2014 | M | 56 | 2 | 4/24/2014 | Summer | Heart Rate |
| W0818 | 2010 | F | 48 | 4 | 4/20/2011 | Winter | Heart Rate |
| W0820 | 2010 | F | 57 | 4 | 4/20/2011 | Winter | Heart Rate |
| W0825 | 2010 | F | 47 | 4 | 6/8/2011 | Winter | Heart Rate |
| W0825 | 2011 | F | 50 | 5 | 4/24/2012 | Winter | Heart Rate |
| W0908 | 2011 | M | 60 | 4 | 4/20/2012 | Winter | Heart Rate |
| W0910 | 2010 | M | 55 | 3 | 4/15/2011 | Winter | Heart Rate |
| W1017 | 2011 | F | 56 | 3 | 2/28/2012 | Winter | Heart Rate |
| W1103 | 2011 | M | 30 | 2 | 4/25/2012 | Winter | Heart Rate |
| W1104 | 2011 | F | 30 | 2 | 2/29/2012 | Winter | Heart Rate |
| W1104 | 2012 | F | 52 | 3 | 2/25/2013 | Winter | Heart Rate |
| W1105 | 2011 | F | 32 | 2 | 2/29/2012 | Winter | Heart Rate |
| W1105 | 2012 | F | 55 | 3 | 2/25/2013 | Winter | Heart Rate |
| W1110 | 2012 | F | 53 | 3 | 2/27/2013 | Winter | Heart Rate |
| W1204 | 2012 | M | 40 | 2 | 2/26/2013 | Winter | Heart Rate |
| W1207 | 2012 | M | 54 | 2 | 2/27/2013 | Winter | Heart Rate |
| W1304 | 2014 | F | 45 | 3 | 2/24/2015 | Winter | Heart Rate |
| W1316 | 2014 | M | 43 | 3 | 4/22/2015 | Winter | Heart Rate |
| W1317 | 2014 | M | 46 | 3 | 4/22/2015 | Winter | Heart Rate |
| W1408 | 2014 | F | 53 | 2 | 4/18/2015 | Winter | Heart Rate |
| W1415 | 2014 | F | 28 | 2 | 2/25/2015 | Winter | Heart Rate |
| W0605 | 2011 | F | 67 | 7 | 5/21/2012 | Winter | Heart Rate |
| W0610 | 2012 | F | 75 | 8 | 4/19/2013 | Winter | Heart Rate |
| W0611 | 2011 | F | 82 | 7 | 5/22/2012 | Winter | Heart Rate |
| W0703 | 2011 | F | 101 | 6 | 8/14/2012 | Winter | Heart Rate |
| W0716 | 2012 | F | 87 | 12 | 1/1/2013 | Winter | Heart Rate |
| W0716 | 2013 | F | 87 | 13 | 4/26/2014 | Winter | Heart Rate |
| W0720 | 2011 | F | 105 | 12 | 8/15/2012 | Winter | Heart Rate |
| W0811 | 2011 | M | 102 | 5 | 4/19/2012 | Winter | Heart Rate |
| W0812 | 2010 | M | 74 | 4 | 4/15/2011 | Winter | Heart Rate |
| W0812 | 2011 | M | 93 | 5 | 4/19/2012 | Winter | Heart Rate |
| W0812 | 2012 | M | 113 | 6 | 4/16/2013 | Winter | Heart Rate |
| W0820 | 2011 | F | 62 | 5 | 4/27/2012 | Winter | Heart Rate |
| W0820 | 2012 | F | 62 | 6 | 1/1/2013 | Winter | Heart Rate |
| W0824 | 2010 | M | 74 | 4 | 4/19/2011 | Winter | Heart Rate |
| W0904 | 2011 | F | 63 | 4 | 4/26/2012 | Winter | Heart Rate |
| W0910 | 2011 | M | 100 | 4 | 4/20/2012 | Winter | Heart Rate |
| W1205 | 2014 | F | 75 | 4 | 4/16/2015 | Winter | Heart Rate |
| W1206 | 2014 | F | 91 | 4 | 4/16/2015 | Winter | Heart Rate |
| W1206 | 2015 | F | 120 | 5 | 4/30/2016 | Winter | Heart Rate |
| W1305 | 2014 | F | 72 | 3 | 2/24/2015 | Winter | Heart Rate |
| W1409 | 2014 | M | 64 | 2 | 4/18/2015 | Winter | Heart Rate |
| W0805 | 2011 | M | 207 | 10 | 4/19/2012 | Winter | Heart Rate |
| W0805 | 2012 | M | 215 | 11 | 5/30/2013 | Winter | Heart Rate |
| W0811 | 2012 | M | 134 | 6 | 4/17/2013 | Winter | Heart Rate |
| W0910 | 2012 | M | 139 | 5 | 4/17/2013 | Winter | Heart Rate |
| W1019 | 2011 | M | 233 | 8 | 4/26/2012 | Winter | Heart Rate |
| W1211 | 2014 | M | 157 | 15 | 4/17/2015 | Winter | Heart Rate |
| W0611 | 2011 | F | 55 | 6 | 4/27/2011 | Summer | Body Temperature |
| W0720 | 2011 | F | 56 | 11 | 4/19/2011 | Summer | Body Temperature |
| W0818 | 2010 | F | 58 | 3 | 6/22/2010 | Summer | Body Temperature |
| W0820 | 2011 | F | 57 | 4 | 4/20/2011 | Summer | Body Temperature |
| W0825 | 2010 | F | 53 | 3 | 6/21/2010 | Summer | Body Temperature |
| W0825 | 2011 | F | 47 | 4 | 6/8/2011 | Summer | Body Temperature |
| W0908 | 2011 | M | 51 | 3 | 6/9/2011 | Summer | Body Temperature |
| W0910 | 2010 | M | 56 | 2 | 6/21/2010 | Summer | Body Temperature |
| W0910 | 2011 | M | 55 | 3 | 4/15/2011 | Summer | Body Temperature |
| W1005 | 2011 | M | 22 | 2 | 4/19/2011 | Summer | Body Temperature |
| W1017 | 2011 | F | 28 | 2 | 6/8/2011 | Summer | Body Temperature |
| W1104 | 2012 | F | 29 | 2 | 6/11/2012 | Summer | Body Temperature |
| W1110 | 2012 | F | 29 | 2 | 6/12/2012 | Summer | Body Temperature |
| W1205 | 2014 | F | 59 | 3 | 4/15/2014 | Summer | Body Temperature |
| W1302 | 2014 | M | 52 | 2 | 4/24/2014 | Summer | Body Temperature |
| W1304 | 2014 | F | 33 | 2 | 7/2/2014 | Summer | Body Temperature |
| W1305 | 2014 | F | 49 | 2 | 7/1/2014 | Summer | Body Temperature |
| W1308 | 2014 | M | 39 | 2 | 4/23/2014 | Summer | Body Temperature |
| W1316 | 2014 | M | 42 | 2 | 7/1/2014 | Summer | Body Temperature |
| W1408 | 2014 | F | 25 | 1 | 4/27/2014 | Summer | Body Temperature |
| W1415 | 2014 | F | 17 | 1 | 5/19/2014 | Summer | Body Temperature |
| W0605 | 2011 | F | 73 | 6 | 4/18/2011 | Summer | Body Temperature |
| W0610 | 2012 | F | 62 | 7 | 5/22/2012 | Summer | Body Temperature |
| W0716 | 2012 | F | 87 | 11 | 5/2/2012 | Summer | Body Temperature |
| W0720 | 2012 | F | 105 | 12 | 8/15/2012 | Summer | Body Temperature |
| W0811 | 2012 | M | 102 | 5 | 8/14/2012 | Summer | Body Temperature |
| W0812 | 2010 | M | 77 | 3 | 6/21/2010 | Summer | Body Temperature |
| W0812 | 2011 | M | 74 | 4 | 6/7/2011 | Summer | Body Temperature |
| W0812 | 2012 | M | 93 | 5 | 4/19/2012 | Summer | Body Temperature |
| W0820 | 2012 | F | 62 | 5 | 4/27/2012 | Summer | Body Temperature |
| W0824 | 2010 | M | 63 | 3 | 6/21/2010 | Summer | Body Temperature |
| W0904 | 2011 | F | 72 | 3 | 6/9/2011 | Summer | Body Temperature |
| W0904 | 2012 | F | 63 | 4 | 4/26/2012 | Summer | Body Temperature |
| W0910 | 2012 | M | 100 | 4 | 4/20/2012 | Summer | Body Temperature |
| W1206 | 2014 | F | 63 | 3 | 4/15/2014 | Summer | Body Temperature |
| W1206 | 2015 | F | 91 | 4 | 4/16/2015 | Summer | Body Temperature |
| W9615 | 2011 | F | 90 | 16 | 4/20/2011 | Summer | Body Temperature |
| W0718 | 2011 | M | 171 | 11 | 4/14/2011 | Summer | Body Temperature |
| W0802 | 2011 | M | 152 | 8 | 4/14/2011 | Summer | Body Temperature |
| W0805 | 2011 | M | 200 | 9 | 4/18/2011 | Summer | Body Temperature |
| W0805 | 2012 | M | 207 | 10 | 4/19/2012 | Summer | Body Temperature |
| W0910 | 2015 | M | 164 | 7 | 4/18/2015 | Summer | Body Temperature |
| W1001 | 2011 | M | 153 | 11 | 4/14/2011 | Summer | Body Temperature |
| W1019 | 2011 | M | 206 | 7 | 4/15/2011 | Summer | Body Temperature |
| W1019 | 2012 | M | 233 | 8 | 4/26/2012 | Summer | Body Temperature |
| W1020 | 2011 | M | 178 | 7 | 4/14/2011 | Summer | Body Temperature |
| W1314 | 2015 | M | 128 | 6 | 4/16/2015 | Summer | Body Temperature |
| W0611 | 2011 | F | 82 | 7 | 4/27/2011 | Winter | Body Temperature |
| W0716 | 2013 | F | 87 | 13 | NA | Winter | Body Temperature |
| W0805 | 2011 | M | 207 | 10 | 4/18/2011 | Winter | Body Temperature |
| W0805 | 2012 | M | 215 | 11 | 4/19/2012 | Winter | Body Temperature |
| W0811 | 2011 | M | 102 | 5 | 4/15/2011 | Winter | Body Temperature |
| W0811 | 2012 | M | 134 | 6 | 8/14/2012 | Winter | Body Temperature |
| W0812 | 2010 | M | 74 | 4 | 6/21/2010 | Winter | Body Temperature |
| W0812 | 2011 | M | 93 | 5 | 6/7/2011 | Winter | Body Temperature |
| W0812 | 2012 | M | 113 | 6 | 4/19/2012 | Winter | Body Temperature |
| W0818 | 2010 | F | 48 | 4 | 6/22/2010 | Winter | Body Temperature |
| W0820 | 2010 | F | 57 | 4 | 6/22/2010 | Winter | Body Temperature |
| W0820 | 2011 | F | 62 | 5 | 4/20/2011 | Winter | Body Temperature |
| W0824 | 2010 | M | 74 | 4 | 6/21/2010 | Winter | Body Temperature |
| W0825 | 2010 | F | 47 | 4 | 6/21/2010 | Winter | Body Temperature |
| W0825 | 2011 | F | 50 | 5 | 6/8/2011 | Winter | Body Temperature |
| W0904 | 2011 | F | 63 | 4 | 6/9/2011 | Winter | Body Temperature |
| W0908 | 2011 | M | 60 | 4 | 6/9/2011 | Winter | Body Temperature |
| W0910 | 2010 | M | 55 | 3 | 6/21/2010 | Winter | Body Temperature |
| W0910 | 2011 | M | 100 | 4 | 4/15/2011 | Winter | Body Temperature |
| W0910 | 2012 | M | 139 | 5 | 4/20/2012 | Winter | Body Temperature |
| W1017 | 2011 | F | 56 | 3 | 6/8/2011 | Winter | Body Temperature |
| W1019 | 2011 | M | 233 | 8 | 4/15/2011 | Winter | Body Temperature |
| W1103 | 2011 | M | 30 | 2 | 4/18/2011 | Winter | Body Temperature |
| W1104 | 2011 | F | 30 | 2 | 4/20/2011 | Winter | Body Temperature |
| W1104 | 2012 | F | 52 | 3 | 6/11/2012 | Winter | Body Temperature |
| W1105 | 2011 | F | 32 | 2 | 4/20/2011 | Winter | Body Temperature |
| W1105 | 2012 | F | 55 | 3 | 6/11/2012 | Winter | Body Temperature |
| W1110 | 2012 | F | 53 | 3 | 6/12/2012 | Winter | Body Temperature |
| W1204 | 2012 | M | 40 | 2 | 5/2/2012 | Winter | Body Temperature |
| W1205 | 2014 | F | 75 | 4 | 4/15/2014 | Winter | Body Temperature |
| W1207 | 2012 | M | 54 | 2 | 5/3/2012 | Winter | Body Temperature |
| W1304 | 2014 | F | 45 | 3 | 7/2/2014 | Winter | Body Temperature |
| W1305 | 2014 | F | 72 | 3 | 7/1/2014 | Winter | Body Temperature |
| W1316 | 2014 | M | 43 | 3 | 7/1/2014 | Winter | Body Temperature |
| W1317 | 2014 | M | 46 | 3 | 7/1/2014 | Winter | Body Temperature |
| W1408 | 2014 | F | 53 | 2 | 4/27/2014 | Winter | Body Temperature |
| W1409 | 2014 | M | 64 | 2 | 4/27/2014 | Winter | Body Temperature |
| W1415 | 2014 | F | 28 | 2 | 5/19/2014 | Winter | Body Temperature |

| **Table S2: Overview over monthly averaged body temperature and heart rate.**  Month = Month of the year, Body size category (Size) = 3 levels of small (S) bears (< 60 kg), medium sized (M) bears (60 – 120 kg) and large (L) bears (> 120 kg), T_B_ = monthly averaged body temperature over all bears and body size categories, sd TB = monthly averaged standard deviation over all bears and body size categories, mean HR = monthly averaged daytime heart rate, sd HR = standard deviation of daytime heart rate, n = number of observations (data points) | | | | | | | | | | |
| --- | --- | --- | --- | --- | --- | --- | --- | --- | --- | --- |
| Month | Size | T_b_ | SE | n |  | Month | Size | HR | SE | n |
| January | S | 33.1 | 0.8 | 1488 |  | January | S | 16.2 | 5.2 | 1736 |
| January | M | 33.8 | 0.7 | 496 |  | January | M | 17.9 | 6.5 | 1240 |
| January | L | 34.3 | 0.5 | 372 |  | January | L | 14.7 | 3.0 | 558 |
| February | S | 33.6 | 1.0 | 1362 |  | February | S | 16.7 | 6.2 | 1570 |
| February | M | 34.2 | 0.8 | 454 |  | February | M | 17.5 | 6.7 | 1142 |
| February | L | 34.8 | 0.5 | 334 |  | February | L | 15.3 | 2.6 | 514 |
| March | S | 35.1 | 1.1 | 1488 |  | March | S | 24.3 | 8.9 | 1676 |
| March | M | 35.5 | 0.8 | 496 |  | March | M | 23.5 | 12.4 | 1302 |
| March | L | 36.3 | 0.5 | 310 |  | March | L | 24.8 | 9.3 | 558 |
| April | S | 36.1 | 1.2 | 1226 |  | April | S | 44.5 | 18.1 | 1444 |
| April | M | 36.9 | 0.3 | 322 |  | April | M | 38.2 | 14.0 | 1146 |
| April | L | 36.9 | 0.3 | 266 |  | April | L | 45.4 | 9.8 | 540 |
| May | S | 37.4 | 0.3 | 706 |  | May | S | 70.4 | 17.1 | 1692 |
| May | M | 37.2 | 0.3 | 496 |  | May | M | 54.4 | 12.4 | 1366 |
| May | L | 37.1 | 0.3 | 558 |  | May | L | 52.0 | 10.3 | 848 |
| June | S | 37.5 | 0.3 | 1038 |  | June | S | 75.8 | 15.4 | 1796 |
| June | M | 37.4 | 0.3 | 556 |  | June | M | 58.9 | 10.2 | 1408 |
| June | L | 37.0 | 0.4 | 540 |  | June | L | 53.1 | 10.3 | 840 |
| July | S | 37.7 | 0.2 | 1548 |  | July | S | 84.3 | 14.7 | 1854 |
| July | M | 37.4 | 0.2 | 682 |  | July | M | 61.8 | 9.4 | 1554 |
| July | L | 37.1 | 0.3 | 558 |  | July | L | 55.3 | 9.8 | 930 |
| August | S | 37.8 | 0.2 | 1532 |  | August | S | 89.3 | 13.6 | 1834 |
| August | M | 37.5 | 0.3 | 640 |  | August | M | 69.3 | 9.3 | 1542 |
| August | L | 37.2 | 0.3 | 528 |  | August | L | 60.4 | 9.5 | 818 |
| September | S | 37.8 | 0.2 | 1440 |  | September | S | 83.7 | 11.7 | 1680 |
| September | M | 37.5 | 0.3 | 482 |  | September | M | 70.4 | 9.1 | 1212 |
| September | L | 37.3 | 0.3 | 326 |  | September | L | 58.8 | 8.6 | 540 |
| October | S | 37.1 | 0.9 | 1488 |  | October | S | 67.4 | 17.0 | 1736 |
| October | M | 37.2 | 0.4 | 496 |  | October | M | 56.9 | 15.5 | 1240 |
| October | L | 36.8 | 0.7 | 310 |  | October | L | 49.9 | 14.8 | 558 |
| November | S | 34.8 | 1.3 | 1440 |  | November | S | 28.0 | 18.3 | 1680 |
| November | M | 35.5 | 1.0 | 480 |  | November | M | 26.3 | 15.7 | 1200 |
| November | L | 34.8 | 0.8 | 324 |  | November | L | 21.8 | 12.6 | 540 |
| December | S | 33.5 | 0.9 | 1488 |  | December | S | 17.3 | 7.7 | 1736 |
| December | M | 34.2 | 0.8 | 496 |  | December | M | 18.2 | 6.9 | 1240 |
| December | L | 34.3 | 0.4 | 372 |  | December | L | 14.3 | 2.9 | 558 |
| January | S | 33.1 | 0.8 | 1488 |  | January | S | 16.2 | 5.2 | 1736 |

| **Table S3: AIC model selection table for heart rate during hibernation on the random and fixed effect structures.** YID = combination of bear ID and year. Doy = day of the year. Brown bear spring body mass in kg at capture after hibernation. Models included in the model selection for fixed effects included the highest ranked random structure. | | | | |
| --- | --- | --- | --- | --- |
| Model | dLogLik | ΔAIC | df | Weight |
| **Random structure** |  |  |  |  |
| *random intercept and slope*  YID | 2974.3 | 0.0 | 109.5 | 1 |
| *random intercept and slope*  Bear ID | 2301.4 | 1300.5 | 86.8 | <0.001 |
| *random intercept*  YID | 2179.6 | 1478.0 | 53.8 | <0.001 |
| *random intercept*  Bear ID nested in Year | 2013.4 | 1821.4 | 59.3 | <0.001 |
| *random intercept*  Bear ID | 1942.7 | 1954.5 | 55.1 | <0.001 |
| *random intercept*  Year | 0.0 | 5773.7 | 22 | <0.001 |
| **Fixed effects** |  |  |  |  |
| te(doy, Spring body mass) | 76.8 | 0.0 | 109.3 | 1 |
| s(doy) | 46.3 | 48.5 | 103 | <0.001 |
| Null model | 0.0 | 128.9 | 97 | <0.001 |

| **Table S4: AIC model selection table for body temperature during hibernation on the random and fixed effect structures.** YID = combination of bear ID and year. Doy = day of the year. Brown bear spring body mass in kg at capture after hibernation. Models included in the model selection for fixed effects included the highest ranked random structure. | | | | |
| --- | --- | --- | --- | --- |
| Model | dLogLik | ΔAIC | df | Weight |
| **Random structure** |  |  |  |  |
| *random intercept and slope*  YID | 747.6 | 0.0 | 84.9 | 1 |
| *random intercept and slope*  Bear ID | 678.5 | 115.0 | 73.3 | <0.001 |
| *random intercept*  YID | 588.4 | 261.6 | 56.4 | <0.001 |
| *random intercept*  Bear ID nested in Year | 531.6 | 362.2 | 50 | <0.001 |
| *random intercept*  Bear ID | 531.6 | 362.2 | 50 | <0.001 |
| *random intercept*  Year | 0.0 | 1372.5 | 23.5 | <0.001 |
| **Fixed effects** |  |  |  |  |
| te(doy, Spring body mass) | 284.8 | 0.0 | 84.5 | 1 |
| s(doy) | 246.2 | 64.3 | 78.1 | <0.001 |
| Null model | 0.0 | 544.9 | 72.2 | <0.001 |

| **Table S5: AIC model selection table for heart rate during summer and during day time on the random and fixed effect structures.** YID = combination of bear ID and year. Doy = day of the year. Brown bear spring body mass in kg at capture after hibernation. Models included in the model selection for fixed effects included the highest ranked random structure. | | | | |
| --- | --- | --- | --- | --- |
| Model | dLogLik | ΔAIC | df | Weight |
| **Random structure** |  |  |  |  |
| *random intercept and slope*  YID | 1589.9 | 0.0 | 153 | 1 |
| *random intercept and slope*  Bear ID | 1088.3 | 893.9 | 98.4 | <0.001 |
| *random intercept*  YID | 1042.3 | 971.0 | 90.8 | <0.001 |
| *random intercept*  Bear ID nested in Year | 852.6 | 1304.7 | 68 | <0.001 |
| *random intercept*  Bear ID | 766.8 | 1467.5 | 63.6 | <0.001 |
| *random intercept*  Year | 0.0 | 2931.5 | 28.8 | <0.001 |
| **Fixed effects** |  |  |  |  |
| te(doy, Spring body mass) | 134.5 | 0.0 | 152.7 | 0.981 |
| s(doy) | 124.6 | 7.9 | 146.8 | 0.019 |
| Null model | 0.0 | 246.7 | 141.6 | <0.001 |

| **Table S6: AIC model selection table for heart rate during summer and during night time on the random and fixed effect structures.** YID = combination of bear ID and year. Doy = day of the year. Brown bear spring body mass in kg at capture after hibernation. Models included in the model selection for fixed effects included the highest ranked random structure. | | | | |
| --- | --- | --- | --- | --- |
| Model | dLogLik | ΔAIC | df | Weight |
| **Random structure** |  |  |  |  |
| *random intercept and slope*  YID | 2615.6 | 0.0 | 156.6 | 1 |
| *random intercept and slope*  Bear ID | 1885.3 | 1349.9 | 101.3 | <0.001 |
| *random intercept*  YID | 1665.1 | 1771.5 | 91.8 | <0.001 |
| *random intercept*  Bear ID nested in Year | 1398.3 | 2257.2 | 67.9 | <0.001 |
| *random intercept*  Bear ID | 1291.0 | 2462.3 | 63.2 | <0.001 |
| *random intercept*  Year | 0.0 | 4976.0 | 29 | <0.001 |
| **Fixed effects** |  |  |  |  |
| te(doy, Spring body mass) | 586.3 | 0.0 | 156.4 | 1 |
| s(doy) | 369.3 | 419.7 | 149.2 | <0.001 |
| Null model | 0.0 | 1143.9 | 142 | <0.001 |

| **Table S7: AIC model selection table for inactive summer body temperature on the random and fixed effect structures.** YID = combination of bear ID and year. Doy = day of the year. Brown bear spring body mass in kg at capture after hibernation. Models included in the model selection for fixed effects included the highest ranked random structure. | | | | |
| --- | --- | --- | --- | --- |
| Model | dLogLik | ΔAIC | df | Weight |
| **Random structure** |  |  |  |  |
| *random intercept and slope*  YID | 2536.5 | 0.0 | 105.8 | 1 |
| *random intercept and slope*  Bear ID | 2142.1 | 759.2 | 91.1 | <0.001 |
| *random intercept*  YID | 1951.6 | 1091.8 | 66.9 | <0.001 |
| *random intercept*  Bear ID nested in Year | 1799.5 | 1389.1 | 63.5 | <0.001 |
| *random intercept*  Bear ID | 1647.3 | 1683.6 | 58.5 | <0.001 |
| *random intercept*  Year | 0.0 | 4919.6 | 29.1 | <0.001 |
| **Fixed effects** |  |  |  |  |
| te(doy, Spring body mass) | 373.4 | 0.0 | 105.7 | 1 |
| s(doy) | 135.6 | 462.8 | 99.3 | <0.001 |
| Null model | 0.0 | 719.7 | 92.2 | <0.001 |

| **Table S8: Summary statistics for the generalized additive mixed models (GAMM).** Estimates are in °C for the brown bear body temperature models and in beats per minute for the HR models. Doy = day of the year. Te(doy : body mass) is fitted as an interaction like term using a tensor smooth product, allowing the effect of the body mass varying over time. S(YID, “re”) and s(YID, doy, “re”) represent the random intercept and slope in the model. Estimated degree of freedom and referred degree of freedom with p-values <0.05 indicate time dependent nonlinear effects. | | | | |
| --- | --- | --- | --- | --- |
| **HR Winter day** |  |  |  |  |
|  | **Estimate** | **SE** | **T** | **P** |
| **Intercept** | 16.1 | 1.7 | 9.4 | <0.001 |
|  | **Edf.** | **Ref. df.** | **F** | **P** |
| **te(** **doy : body mass)** | 13.53 | 46 | 4.6 | <0.001 |
| **s(YID, “re”)** | 45.22 | 47 | 133583.9 | <0.001 |
| **s(YID, doy, “re”)** | 43.52 | 47 | 129873.7 | <0.001 |
| **Adjusted R^2^** | 0.77 |  |  |  |
| **T_b_ Winter** |  |  |  |  |
|  | **Estimate** | **SE** | **T** | **P** |
| **Intercept** | 33.5 | 0.2 | 223.6 | <0.001 |
|  | **Edf.** | **Ref. df.** | **F** | **P** |
| **te(** **doy: body mass)** | 18.4 | 20.6 | 16.6 | <0.001 |
| **s(YID, “re”)** | 28.8 | 36 | 1697.5 | <0.001 |
| **s(YID, doy, “re”)** | 24.2 | 36 | 1446.5 | <0.001 |
| **Adjusted R^2^** | 0.63 |  |  |  |
| **HR Summer day** |  |  |  |  |
|  | **Estimate** | **SE** | **T** | **P** |
| **Intercept** | 70.3 | 0.8 | 82.5 | <0.001 |
|  | **Edf.** | **Ref. df.** | **F** | **P** |
| **te(** **doy : body mass)** | 19.4 | 21.6 | 45.3 | <0.001 |
| **s(YID, “re”)** | 44.3 | 71 | 106.6 | <0.001 |
| **s(YID, doy, “re”)** | 27.9 | 71 | 65.1 | <0.001 |
| **Adjusted R^2^** | 0.78 |  |  |  |
| **HR Summer night** |  |  |  |  |
|  | **Estimate** | **SE** | **T** | **P** |
| **Intercept** | 77.3 | 7.9 | 9.6 | <0.001 |
|  | **Edf.** | **Ref. df.** | **F** | **P** |
| **te(** **doy : body mass)** | 15.9 | 17.8 | 30 | <0.001 |
| **s(YID, “re”)** | 64.8 | 71 | 429929 | <0.001 |
| **s(YID, doy, “re”)** | 63.9 | 71 | 422323 | <0.001 |
| **Adjusted R^2^** | 0.71 |  |  |  |
| **T_b_ Summer** |  |  |  |  |
|  | **Estimate** | **SE** | **T** | **P** |
| **Intercept** | 37.5 | 0.1 | 533.7 | <0.001 |
|  | **Edf.** | **Ref. df.** | **F** | **P** |
| **te(** **doy**  **: body mass)** | 17.1 | 19.4 | 13.6 | <0.001 |
| **s(YID, “re”)** | 33.8 | 45 | 17164.7 | <0.001 |
| **s(YID, doy, “re”)** | 31.6 | 45 | 15642.9 | <0.001 |
| **Adjusted R^2^** | 0.39 |  |  |  |

| **Table S9: AICc model selection table for the start of hibernation.** Brown bear spring body mass in kg at capture after hibernation, winter capture is a factor indicating if the bear was captured during hibernation, year a factor of the winter the data was collected and sex of the bear. Models within ΔAICc 2 were averaged for interpretation. | | | | | |
| --- | --- | --- | --- | --- | --- |
| Model | K | AICc | AICc | Weight | Cumulative Weight |
| Null model | 1 | 278.05 | 0.00 | 0.65 | 0.65 |
| Spring body mass | 2 | 280.03 | 1.98 | 0.24 | 0.89 |
| Year | 4 | 282.40 | 4.36 | 0.07 | 0.96 |
| Spring body mass + Year | 5 | 284.42 | 6.37 | 0.03 | 0.99 |
| Spring body mass + Sex + Year | 6 | 286.70 | 8.65 | 0.01 | 1.00 |

| **Table S10: AICc model selection table for the hibernation duration.** Brown bear spring body mass in kg at capture after hibernation, winter capture is a factor indicating if the bear was captured during hibernation, year a factor of the winter the data was collected and sex of the bear. Models within Δ AICc 2 were averaged for interpretation. | | | | | |
| --- | --- | --- | --- | --- | --- |
| **Model** | **K** | **AICc** | **AICc** | **Weight** | **Cumulative Weight** |
| Spring body mass | 2 | 293.16 | 0 | 0.44 | 0.44 |
| Spring body mass + Winter capture | 3 | 293.40 | 0.24 | 0.39 | 0.83 |
| Spring body mass + Winter capture + Year | 6 | 295.32 | 2.16 | 0.15 | 0.98 |
| Winter capture | 2 | 299.88 | 6.72 | 0.02 | 1.00 |
| Year + Winter capture | 5 | 303.56 | 10.40 | 0.00 | 1.00 |
| Null model | 1 | 307.40 | 14.24 | 0.00 | 1.00 |
| Year | 4 | 310.24 | 17.08 | 0.00 | 1.00 |

| **Table S11: AICc model selection table for the end of hibernation.** Brown bear spring body mass in kg at capture after hibernation, winter capture is a factor indicating if the bear was captured during hibernation, year a factor of the winter the data was collected and sex of the bear. Models within Δ AICc 2 were averaged for interpretation. | | | | | |
| --- | --- | --- | --- | --- | --- |
| Model | K | AICc | AICc | Weight | Cumulative Weight |
| Spring body mass + Winter capture | 3 | 255.40 | 0.00 | 0.51 | 0.51 |
| Spring body mass | 2 | 255.58 | 0.19 | 0.47 | 0.98 |
| Winter capture | 2 | 262.86 | 7.46 | 0.01 | 0.99 |
| Spring body mass + Winter capture + Year | 6 | 263.49 | 8.10 | 0.01 | 1.00 |
| Year + Winter capture | 5 | 267.90 | 12.57 | 0.00 | 1.00 |
| Null model | 1 | 271.96 | 16.57 | 0.00 | 1.00 |
| Year | 4 | 278.09 | 22.70 | 0.00 | 1.00 |
